# Detection of the pediocin gene *pedA *in strains from human faeces by real-time PCR and characterization of *Pediococcus acidilactici *UVA1

**DOI:** 10.1186/1472-6750-7-55

**Published:** 2007-09-12

**Authors:** Sophie Mathys, Ueli von Ah, Christophe Lacroix, Ernö Staub, Raffaella Mini, Tania Cereghetti, Leo Meile

**Affiliations:** 1 Laboratory of Food Biotechnology, Institute of Food Science and Nutrition, Swiss Federal Institute of Technology (ETH), Zurich, Switzerland

## Abstract

**Background:**

Bacteriocin-producing lactic acid bacteria are commonly used as natural protective cultures. Among them, strains of the genus *Pediococcus *are particularly interesting for their ability to produce pediocin, a broad spectrum antimicrobial peptide with a strong antagonistic activity against the food-borne pathogen *Listeria monocytogenes*. Furthermore, there is increasing interest in isolating new bacteriocin-producing strains of human intestinal origin that could be developed for probiotic effects and inhibition of pathogenic bacteria in the gut. In this work, we typed a new strain, co-isolated from baby faeces together with a *Bifidobacterium thermophilum *strain, and characterized its proteinaceous compound with strong antilisterial activity.

**Results:**

The newly isolated strain UVA1 was identified as a *Pediococcus acidilactici *by carbohydrate fermentation profile, growth at 50°C and 16S rDNA sequencing. The partially purified bacteriocin was heat resistant up to 100°C, active over a wide range of pH (2 to 9) and susceptible to proteolytic enzymes. The molecular weight, estimated by SDS-PAGE, was similar to that of pediocin AcH/PA-1 (4.5 kDa). *P. acidilactici *UVA1 harboured a 9.5-kb plasmid that could be cured easily, which resulted in the loss of the antimicrobial activity. Southern hybridization using the DIG-labelled *pedA*-probe established that the bacteriocin gene was plasmid-borne as for all pediocin described so far. Nucleotide sequence of the whole operon (3.5 kb) showed almost 100 % similarity to the pediocin AcH/PA-1 operon. The mRNA transcript for *pedA *could be detected in *P. acidilactici *UVA1 but not in the cured derivative, confirming the expression of the *pedA*-gene in UVA1. Using a new real-time PCR assay, eleven out of seventeen human faecal samples tested were found to contain *pedA*-DNA.

**Conclusion:**

We identified and characterised the first pediocin produced by a human intestinal *Pediococcus acidilactici *isolate and successfully developed a new real-time PCR assay to show the large distribution of *pedA*-containing strains in baby faecal samples.

## Background

The lactic acid bacteria are an inhomogeneous group which includes among others the genera *Pediococcus*, *Enterococcus*, *Lactococcus*, *Lactobacillus *and *Streptococcus*. Among the *Pediococcus *group, *P. acidilactici *and *P. pentosaceus *are widely used for fermentation of food products like vegetable and meat products. By production of organic acids, resulting in pH decrease, they contribute to control the microbial succession during fermentation. They also act as protective cultures preventing the growth of food-borne pathogens such as *Listeria monocytogenes *or *Staphylococcus aureus *[1] and by doing this, they extend storage life and enhance safety of food products [2,3]. Beside production of classical antimicrobial compounds such as organic acids or hydrogen peroxide, the protective effect also results from the production of bacteriocins [4]. Bacteriocins are ribosomally synthesized, small, heat-stable antimicrobial peptides produced by bacteria. Pediocin AcH/PA-1 is the most studied class IIa bacteriocin (non modified, nonlantibiotic peptides) and has potential for use as food preservative, due to its strong antilisterial activity [5]. Since amino-acid sequence determination of the pediocin AcH/PA-1 in 1992 [6,7], several pediocin-producing *P. acidilactici *and *P. pentosaceus *strains have been screened from a large variety of plants and fruits [8], but also from the gastrointestinal tract of poultry, ducks and other animals [9-11]. Millette *et al*. [12] recently isolated a strain of *Pediococcus acidilactici *from human faeces which produces an unidentified antimicrobial proteinaceous compound. To our knowledge, no pediocin-producing *Pediococcus *has been isolated so far from human faeces and it is therefore unknown to which extent these strains are widespread in the human intestinal microbiota and contribute to the microbial balance of the complex gut ecosystem.

Real-time PCR is a very sensitive and rapid molecular method for the detection of specific genes in complex samples. It is particularly suitable for measuring non cultivable bacteria, because detection is independent of growth conditions of the target organism [13]. It has for example recently been used successfully on faecal samples to assess the survival of *Lactococcus lactis *subsp. *cremoris *FC after transit through the gastrointestinal tract [14] or to detect the presence of noroviruses in clinical stool samples [15].

In this work, we report the identification of a new bacteriocin-producing strain of *Pediococcus acidilactici *(UVA1), which was co-isolated from baby faeces with the bacteriocinogenic strain *Bifidobacterium thermophilum *RBL67 [16]. The biochemical and genetic characterization of the antimicrobial compound was performed. The distribution of *pedA*-containing strains in human faeces was also investigated using a newly designed real-time PCR assay targeting the pediocin structural gene.

## Methods

### Bacterial strains, and growth conditions and plasmid-curing

*Lactobacillus paracasei *subsp. *paracasei *DSM5622^T^, *Pediococcus acidilactici *DSM 20284^T ^and *Pediococcus pentosaceus *DSM 20336^T ^(obtained from DSMZ GmbH, Braunschweig, Germany) were used as reference strains for analysis of the carbohydrate fermentation profile. *Listeria ivanovii *HPB28 (obtained from the Health Protection Branch, Health and Welfare, Ottawa, Canada) was used as indicator strain for the detection of pediocin activity [17]. *P. acidilactici *UL5 (own culture collection) was used as pediocin producer control [17]. *Pediococcus acidilactici *UVA1 was previously isolated from human baby faeces as a stable consortium with the *Bifidobacterium *strain RBL67 [16], which was recently identified as *Bifidobacterium thermophilum*. Strain UVA1 was purified by subsequent selective plating and analysis of single colonies by genus-specific probes (von Ah, unpublished). A non-bacteriocin producing derivative of *P. acidilactici *UVA1, named bac-, was obtained after curing by novobiocin treatment (Sigma-Aldrich Chemie GmbH, Buchs, Switzerland, 0.1 μg ml^-1^) as already described [18]. Sixty colonies were screened by the overlay method for absence of antimicrobial activity against *L. ivanovii *HPB28 and four mutants producing no inhibition halo were detected. One of them was purified on MRSC agar, consisting of MRS ([19] Biolife, Milan, Italy) supplemented with 0.05 % L- cysteine hydrochloride and 1.5 % agar, and propagated in MRSC broth. The cell-free supernatant was checked for the absence of antilisterial activity and absence of the *pedA*-gene was confirmed by PCR.

All lactic acid bacteria were routinely grown overnight in MRSC medium, with incubation at 37°C in anaerobic jars with an atmosphere generation system (Oxoid AnaeroGen TM, Basel, Switzerland). *L. ivanovii *HPB28 was propagated in TSY medium consisting of tryptic soy broth (Oxoid) containing 0.6 % (w/v) yeast extract (Merck, Darmstadt, Germany) overnight at 30°C. For agar plates and soft-agar, the media were supplemented with 1.5 % and 0.75 % (w/v) agar, respectively. Bacterial stocks were stored at -80°C in appropriate media supplemented with 33 % (v/v) glycerol and subcultured three times at one day intervals on fresh agar plates before use. Cultures were routinely checked under a light microscope for contamination.

### Carbohydrate fermentation profile

The carbohydrate fermentation profile of strain UVA1 was determined using API 50 fermentation strips (BioMérieux, Marcy l'Etoile, France). A 2-ml volume of an overnight culture (16 h) was centrifuged at 14'000 × g for 5 min at 4°C. The pellet was washed once and resuspended in 1 ml of sterile, double distilled water. The suspension was then added to 5 ml of CHL 50 medium (prepared according to the manufacturer's instructions). Each tube of the API CHL50 strips was then inoculated with 100 μl of the bacterial suspension in CHL50 medium and sealed with sterile paraffin. The strips were incubated at 37°C under anaerobic conditions, the results were visually assessed after 24, 48 and 72 h and analysed with the APILAB+ software version 3.3.3 according to the manufacturer's instructions. *P. acidilactici *UL5, *P. acidilactici *DSM20284^T^, *P. pentosaceus *DSM20336^T^, and *L. paracasei *subsp. *paracasei *DSM5622^T ^were used as reference strains. All tests were repeated three times.

### Determination of growth at 50°C

Strain UVA1 was incubated at 50°C in 20 ml MRSC medium inoculated with 1 % of an overnight culture. *P. acidilactici *UL5 and DSM20284^T ^and *P. pentosaceus *DSM20336^T ^were used as controls. The optical density at 600 nm was measured with an Uvikon 810P photometer (Kontron Instruments, Rotkreuz, Switzerland) after 4, 24 and 96 h. The ability to grow at 50°C was positive if OD_600 _exceeded 0.4 after 96 h. OD_600 _of 0.4 was set as the limit of growth and corresponded to twice the value of the OD of freshly inoculated medium. All tests were performed twice.

### Inhibition assay

Antibacterial activity was assessed by the agar-well diffusion method. Briefly, 25 ml of soft-agar (heated at 45°C) was inoculated with 0.1 % of an overnight culture of the indicator strain *L. ivanovii *HBP28, poured into a Petri dish and allowed to set for 30 min at room temperature. Holes (diameter of 7 mm) were then punched in the agar and filled with 80 μl of sample. The plates were incubated at 4°C for 30 min to allow bacteriocin diffusion and overnight at 30°C for growth of the indicator strain. The diameter of the inhibition zone was measured.

### Effect of temperature, pH, enzymes and other agents on bacteriocin activity

Cell-free supernatant (CFS) was obtained after centrifugation at 13'000 g for 10 min at 4°C of a 16-h culture in MRSC at pH 6 and 37°C. The supernatant was heated 5 min at 95°C. The effect of temperature on the antibacterial activity was tested after heating at 121°C for 15 min in an autoclave and at 100°C for 60 and 40 min using a water-bath. The effect of pH was tested by adjusting the pH of the CFS to values in a range from 2 to 11 using either 1 M HCl or 1 M NaOH. Residual activity was measured by the agar-well diffusion method, after one day, one week and one month storage at 4°C. To test the sensitivity to proteases and other agents, the CFS was incubated for 2 h at 37°C in the presence of 1 mg ml^-1 ^chymotrypsin, pepsin, protease, proteinase K, trypsin or lysozyme or 1 % SDS, urea, catalase, RNAse A, Tween 20, Tween 80, Triton-X or 2, 5 or 10 mM EDTA. For enzyme denaturation, the samples were finally heated at 95°C for 5 min and residual activity was measured by the agar-well diffusion method. All enzymes and other chemicals were purchased from Sigma-Aldrich Chemie GmbH (Buchs, Switzerland), except proteinase K and trypsin, which were obtained from Applichem (Darmstadt, Germany). Residual activity was defined as the ratio of the diameter of the halo produced by the treated sample compared to the untreated control and expressed in percentage. All assays were performed twice

### Molecular weight determination

The bacteriocin produced by *P. acidilactici *UVA1 was partially purified by injecting 300 ml CFS at a rate of 1 ml min^-1 ^in a 60-ml SP Sepharose column connected to a FPLC chromatography system (Ätka Purifier 10, Amersham, Otelfingen, Switzerland). The column was first equilibrated with 10 column volumes of 5 mM ammonium acetate buffer (pH 5.0), 5-ml fractions were collected in a fraction collector (Frac-950, Amersham) and the bacteriocin-like activity was eluted with 0.45 M NaCl in the same buffer. The CFS and active fractions after FPLC were 10-fold concentrated by ultrafiltration (cutoff of 3 kDa) and 15 μl of the samples were loaded on two parallel SDS gels, along with 10 μl of Polypeptide SDS-PAGE molecular weight standard (BioRad Laboratories AG, Reinach, Switzerland). The gels were prepared according to Schägger and Jagow [20] and consisted of a 10 % acrylamide-bisacrylamide stacking gel and a 16.5 % separating gel. Separation was done with constant voltage (100 V) for 2 h 30 using a vertical slab gel apparatus (BioRad Laboratories). One of the gels was stained with Coomassie brilliant blue R250 (LK Bromma, Villeneuve-la-Garenne, France) and the other was used for activity detection: the gel was first soaked for 2 h in fixation solution (20 % isopropanol, 10 % acetic acid) and rinsed overnight in HPLC-grade water before being overlaid with 25 ml soft TSY agar inoculated with 0.1 % of an overnight culture of *L. ivanovii *HBP28. The molecular weight was estimated by comparison of the mobility of the inhibition zone to that of the molecular weight marker run simultaneously. The whole procedure was repeated twice.

### DNA sequencing and PCR conditions

Sequencing of DNA was performed by Microsynth (Balgach, Switzerland) and similarity searches were conducted with the BLAST program from NCBI (version 2.2.15). Primers and probe used in this study are listed in Table 1. They were designed with the program Primer3 [21] and synthesised by Microsynth. The PCR reactions were set up in a total volume of 50 μl containing 2.5 U EuroTaq-DNA-Polymerase (Digitana, Horgen, Switzerland), 1.5 mM magnesium chloride (Digitana), 0.2 mM dNTP's (GE Healthcare), 0.5 μM of each primer and either 2 μl of DNA or 40 μl of cell suspension (prepared by resuspending a single colony in 210 μl of sterile, double distilled water). The 16S rDNA of *P. acidilactici *UVA1 and UL5 were amplified using a slightly modified protocol from Schürch [22]. The annealing temperature for the bak11w/bak4 primers was increased to 62°C. The 16s rDNA sequences of UVA1 and UL5 are deposited at GenBank under accession numbers [GenBank: EF059986] and [GenBank: EF059987], respectively. For the amplification and sequencing of the first 711 bp of the pediocin PA-1 operon, primers P1 and P2 and conditions described by Rodriguez *et al*. [23] were used. The second part of the operon (2864 bp) was amplified with primers pedopF and pedopR, designed on the basis of the reported sequence for pSRQ11 [24]. Amplification conditions were as follow: 2 min at 95°C, 30 cycles of 1 min at 94°C, 35 s at 45°C and 3 min at 72°C and final elongation step 7 min at 72°C. Oligonucleotides pedopF and pedopR as well as pedseq A, B, C and D, designed every 500 bp along the PCR product, were used as sequencing primers. Additionally, a 1009-bp sequence directly upstream and a 1417-bp sequence directly downstream of the operon were amplified and sequenced with primer pairs pedseq L and H and pedseq M and N, respectively. Amplification conditions were: 3 min at 95°C, 30 cycles of 1 min at 95°C, 35 s at 55°C and 2 min at 72°C and final elongation step 7 min at 72°C.

**Table 1:** Primers and probe used in this study

**Primer/probe**	**Target/specificity**	**Sequence (5'-3')**	**Product size (bp)**	**Reference**
Bak11w	16S rDNA	AGT TTG ATC MTG GCT CAG		[35]
Bak4		AGG AGG TGA TCC ARC CGC A		[36]
P1	pedA-pedB	AAA ATA TCT AAC TAA TAC TTG		
P2		TAA AAA GAT ATT TGA CCA AAA	711	[23]
pedopF	pedB-pedD	GGG CGA GTT TAA CAT GCT AGA		
pedopR		TGA TTA TGA ATT AAC CGT GCA	2862	This study
pedseqA	Sequencing of the pediocin operon	CTT GCT CGA TAA TGG TAA		
pedseqB		CTA TCA GGT AAC TGA AAA		
pedseqC		CAC GCT TTT CTG ATG CAA		
pedseqD		TTC TTG ACC CCA TTA GAA		This study
pedseqL	Upstream of operon	AGC AAT TAC AGT CAA CCA TAA CCA T		
pedseqH		ACA GAG CAG GAA TGT TTG CCA CAA G	1009	This study
pedseq M	Downstream of operon	ATT GTC TCC GGC ACA ATG TT		
pedseqN		CAA AGT GGG GAA ACT CGA AA	1417	This study
pedA2RTF	Real-time PCR	GGC CAA TAT CAT TGG TGG TA		
pedA2RTR		ATT GAT TAT GCA AGT GGT AGC C		
TqM-pedA		FAM-ACT TGT GGC AAA CAT TCC TGC TCT GTT GA-TAMRA	100	This study

### Plasmid isolation and Southern blotting

Extrachromosomal DNA elements were extracted from *P. acidilactici *UVA1, UL5 and the bac- mutant using a modified method after Anderson and McKay for small scale plasmid isolation [25]. Shortly, for cell lysis, 9.5 μl mutanolysin (1500 U ml^-1 ^Sigma-Aldrich Chemie GmbH) was added to the lysis solution (solution B) and plasmid DNA was resuspended in 1 × TE buffer. Finally, the RNA was digested with 10 μg RNase A (Sigma-Aldrich Chemie GmbH). The DNA was visualized after electrophoresis on a 0.65 % agarose gel in 1 × TBE at 100 V for 1 h 30. The supercoiled DNA ladder (Promega, Madison WI, USA) was used as size standard. DIG-labelling of the *pedA*-probe (P1-P2 PCR product on *P. acidilactici *UVA1), blotting on nylon membrane, hybridisation (at 42°C) and chemiluminescent detection were conducted with the DIG-High Prime DNA Labeling and Detection Starter Kit II (Roche Diagnostics, Rotkreuz, Switzerland) according to supplier's instructions. Plasmid preparation, blotting and hybridization were performed twice.

### RNA isolation and reverse transcription

The RNA was isolated during the exponential growth-phase of *P. acidilactici *UVA1, bac-, UL5 and DSM 20284^T^, and *P. pentosaceus *DSM 20336^T ^using the RNeasy Mini kit (Qiagen, Basel, Switzerland). The protocol was slightly modified by addition of 20 U mutanolysin in the lysis solution. The samples were finally treated with RNase-free DNAse I (Invitrogen, Basel, Switzerland) for 30 min at 37°C. First strand cDNA synthesis was performed with the Omniscript reverse transcription kit (Qiagen) and 5 μl of the product were used for PCR amplification of a 100 bp-fragment with primers pedA2RTF and pedA2RTR. PCR products were separated on a 2 % agarose gel in 1 × TAE buffer by electrophoresis at 90 V for 2 h 30. The low molecular weight DNA ladder and Tridye 100 bp DNA-ladder (New England BioLabs, Ipswich, MA, USA) were used as size standards.

### Preparation of DNA from faecal samples

Twenty-one human faecal samples were collected in collaboration with the Department of Gastroenterology (Hospital for Sick Children, Zurich, Switzerland). Thirteen faecal samples were collected from children donors aged one month to 3 years and 4 from adults. Faecal samples were collected within 1 h after defecation, placed in anaerobic jars and rapidly transported to our laboratory. They were immediately frozen at -20°C upon arrival, i.e. no more than 3 h after defecation. Total DNA was isolated from 200 mg of each sample using the QIAamp DNA Stool Mini kit (Qiagen) according to the manufacturer's instructions. Before DNA extraction, one faecal sample was autoclaved twice (121°C, 15 min) to obtain a sample free of DNA. Ten aliquots of this sample were spiked with a 10-fold serial dilution of *P. acidilactici *UVA1 (overnight culture in MRSC) at concentrations ranging from 10^9 ^to 10^1 ^bacteria cells per g faeces. The extracted DNA was stored at -20°C.

### Real-time PCR

Primers and TaqMan probe listed in Table 1 were designed based on the *pedA *sequence with the software PrimerExpress 1.5 (Applied Biosystems, Rotkreuz, Switzerland) and synthesized by Microsynth. The TaqMan probe was labeled with 5'-FAM as a fluorescent reporter dye and 3'-TAMRA as a quencher. Their specificity was tested using the BLAST program from NCBI. Reactions were set in a total volume of 25 μl, containing 2.5 μl of faecal DNA extract, 12.5 μl of qPCR MasterMix from Eurogentec (Seraing, Belgium), 0.3 μM of each primer and 0.1 μM of the TaqMan probe. Reactions were run on an ABI PRISM 7700 Sequence Detector (Applied Biosystems, Rotkreuz, Switzerland). The amplification conditions were 2 min at 50°C, 10 min denaturation at 95°C, followed by 45 cycles of 15 sec at 95°C and 1 min at 60°C. The cycle threshold (Ct), corresponding to the number of cycle after which the target-DNA concentration increase become exponential, was monitored. Results were analysed using the SDS 2.1 Software (Applied Biosystems). All reactions were done in triplicate and repeated three times.

## Results

### Phenotypic identification of strain UVA1 as *Pediococcus acidilactici*

A carbohydrate fermentation profile was performed using API 50 CHL strips after 72 h anaerobic incubation at 37°C. Using the APILAB+ software and *P. acidilactici *DSM20284^T^, *P. acidilactici *UL5 (pediocin-producer) and *P. pentosaceus *DSM20336^T ^as control strains, UVA1 was identified as *Pediococcus acidilactici*. Strains UVA1, UL5 and DSM20284^T ^differed from each other. Mannose was fermented by UL5 and DSM20284^T ^but not by UVA1. Saccharose was only fermented by UL5 and DSM20336^T ^whereas D-trehalose was only fermented by DSM20284^T ^and DSM20336^T^. Using optical density measurement at 600 nm after 96 h incubation, strain UVA1, as well as *P. acidilactici *DSM20284^T^and UL5, were shown to grow at 50°C, whereas *P. pentosaceus *DSM20336^T ^didn't.

### Genotypic confirmation of the identity of *P. acidilactici *UVA1

The 16S rDNA sequences of strain UVA1 and *P. acidilactici *UL5 were analysed using the software BioEdit [26]. The assembly of sequence fragments produced a 1492 and 1568 bp sequences for strains UVA1 and UL5, respectively. The sequences, compared with 16S rDNA sequences in the GenBank, showed 99 % similarity with the type strain *Pediococcus acidilactici *DSM20284^T^. The results of 16S rDNA sequencing identified strain UVA1 as a new *Pediococcus acidilactici *strain. Furthermore, the 16S sequences of strains *P. acidilactici *UVA1 and UL5 were also 99% similar, showing a close relationship between these two strains.

### Biochemical characterization of the antimicrobial compound produced by *P. acidilactici *UVA1

Heating the cell-free supernatant (CFS) at 121°C for 15 min destroyed the inhibitory activity against *Listeria ivanovii *HPB28, whereas heat treatments at 100°C for 40 and 60 min only yielded a slight reduction of the activity with 80 and 75 % residual activity, respectively. Activity remained unaffected after one month storage at 4°C at pH values from 2 to 8. At pH 10 and 11, total loss of activity was already observed after 1 week storage and only 60% of the initial activity was still measurable after 1 month at pH 9. Treatment with proteolytic enzymes such as chymotrypsin, pepsin, protease, proteinase K or trypsin resulted in a total loss of activity while lysozyme, catalase and other agents tested had no effect.

### Determination of the molecular weight

**Figure 1:**
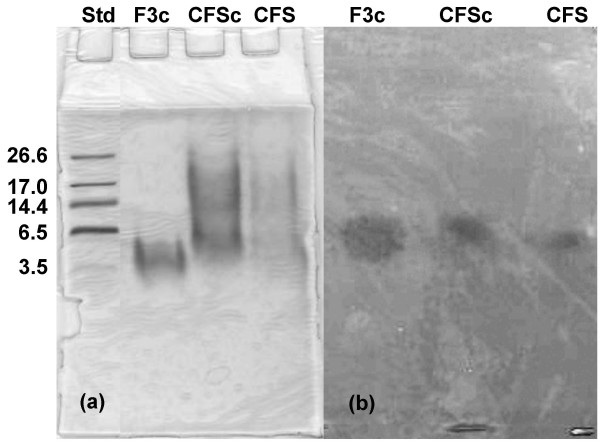
**Molecular weight determination of the bacteriocin produced by strain UVA1**. (a) Coomassie stained SDS-PAGE gel. (b) SDS gel overlaid with the indicator strain *Listeria ivanovii *HPB28. Std: polypeptide molecular weight standard in kDa, F3c: active FPLC-fraction 10-fold concentrated. CFSc: cell-free supernatant of a UVA1 culture, 10-fold concentrated. CFS: cell-free supernatant not concentrated.

As shown in Figure 1, SDS-PAGE of the partially purified bacteriocin (concentrated active FPLC-fraction) showed a diffuse band on the Coomassie-stained gel while a clear inhibition halo was visible on the activity-gel for the concentrated FPLC-fraction as well as for the CFS. The relative mobility of the inhibition zone on the gel overlaid with *Listeria ivanovii *HBP28 was compared to that of the standards stained with Coomassie blue. The apparent molecular weight of the bacteriocin was estimated in the range of 4.5 to 5 kDa. The biochemical properties and the molecular weight described above, as well as the fact that this proteinaceous compound is produced by a *Pediococcus *strain suggest that the anti-*Listeria *compound is a pediocin-like bacteriocin. A genetic approach was used to confirm this assumption.

### Genetic characterization of the bacteriocin

Primers P1 and P2 were used to amplify and sequence a 711-bp fragment (from 91 bp upstream of *pedA *to 33 bp downstream of the translational start of *pedC *[GenBank: M83924]) including the ribosome binding site as well as the -35/-10 region. The remaining sequence of the operon (2862 bp, from 13 bp upstream the end of *pedB *to the end of *pedD*) was amplified with primers pedopF and pedopR (Table 1) and the generated DNA fragment was sequenced. This resulted in a 3473-bp sequence, comprising the four genes constituting the operon including the upstream region of *pedA *(91 bp), presumably containing the regulatory elements of the operon. The whole nucleotide sequence showed more than 99.5 % similarity to the published sequences for *P. acidilactici *K1 [GenBank: AY705375], *P. acidilactici *H [GenBank: U02482], *P. acidilactici *PAC1.0 [GenBank: M83924], *P. pentosaceus *[GenBank: AY316525], *Lactobacillus plantarum *[GenBank: AY316526], *P. parvulus *[GenBank: AY316524], and *Bacillus coagulans *[GenBank: AF300457]. Two sequences of 1009 and 1417 bp, upstream and downstream of the operon, respectively, were also PCR-amplified and sequenced. The resulting 5368-bp sequence was 99 % identical to the pediocin-encoding plasmid pSRQ11 described for *P. acidilactici *PA-1 [27].

### The antimicrobial activity is linked to the presence of the plasmid and the expression of *pedA*

**Figure 2:**
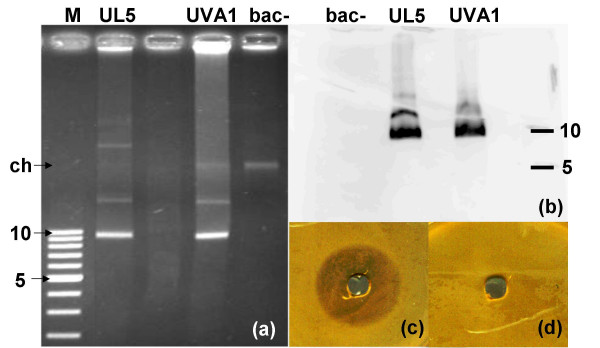
**Localization of the *pedA *gene on the plasmid by curing and Southern blotting**. (a) Agarose gel electrophoresis of plasmid DNA. UVA1: *P. acidilactici *UVA1. UL5: *P. acidilactici *UL5. bac-: cured derivative of UVA1. M. Supercoiled DNA ladder in kb. ch: chromosomal DNA band. (b) Southern blot DNA hybridization of the DIG labeled *pedA*-probe with plasmidic DNA from *P. acidilactici *bac-, UVA1 and UL5. (c) and (d) Agar-well diffusion assay with CFS of cultures of *P. acidilactici *UVA1 (c) and its cured derivative *P. acidilactici *bac- (d).

**Figure 3:**
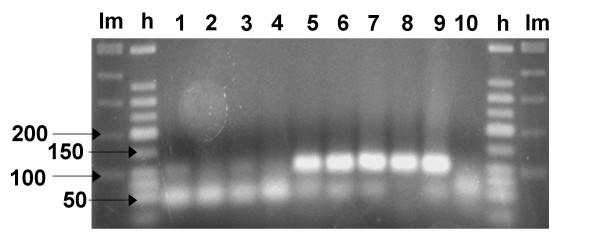
**Transcription analysis of *pedA***. *pedA*-reverse transcription-PCR on cDNA from *P. acidilactici *bac- (1, 2, 3) or UVA1 (7, 8, 9) after 1 h 30, 2 h 30 and 3 h 30 of growth, respectively, and from *P. acidilactici *DSM 20284^T^(4) or UL5 (6) and *P. pentosaceus *DSM 20336^T ^(5) after 2 h 30 of growth. 10: water instead of DNA. lm: low molecular weight DNA ladder (in bp). h: Tridye 100-bp DNA ladder (in bp). Expected product size: 100 bp.

Agarose gel electrophoresis of plasmid DNA preparation from strain UVA1 showed three bands: two extrachromosomal DNA elements at 9.5 kb and at >10 kb and one corresponding to chromosomal DNA, respectively (Figure 2(a)). For strain bac-, a non-inhibitory derivative of *P. acidilactici *UVA1 obtained after plasmid curing, in contrast, only chromosomal DNA was visible. Plasmid DNA prepared from the control pediocin producing strain UL5 exhibited the same bands as detected for UVA1, in addition to two larger extrachromosomal DNA elements. Southern hybridization with the *pedA*-probe (Figure 2(b)) yielded positive signals for the 9.5-kb and the >10-kb extrachromosomal elements present in UL5 and UVA1, but no hybridization occurred for bac-, confirming the presence of the *pedA*-gene on the 9.5-kb plasmid, the band at >10 kb probably being the relaxed circular form of the same plasmid. The presence of the *pedA*-gene on the plasmid was confirmed by an agar-well diffusion assay, where the supernatant of a culture of the bac- strain failed to exhibit inhibition of the indicator strain *Listeria ivanovii *HPB28 (Figure 2(c) and 2(d)). Furthermore, the *pedA*-transcript was detected by reverse-transcription-PCR on cDNA from *P. acidilactici *UVA1 (Figure 3, lanes 7–9) but not from the cured derivative bac- (Figure 3, lanes 1–3), providing the ultimate link between presence of the plasmid-localized genetic determinant and expression of the bacteriocin. The presence of a slight band at 100 bp for the non-plasmid containing strains (Figure 3, lanes 1–4) could not be explained, but the very weak intensity compared to lanes 7 to 9 does not represent a positive signal. A second PCR, performed with primers P1 and P2 (Table 1), which encompass the regulatory region upstream of the transcriptional start, resulted in no amplification in samples using cDNA as template, allowing us to exclude genomic DNA contamination in the RNA preparation of UVA1, UL5 and *P. pentosaceus *DSM 20336^T ^(data not shown).

### Distribution of *pedA*-containing strains in human faecal samples

**Figure 4:**
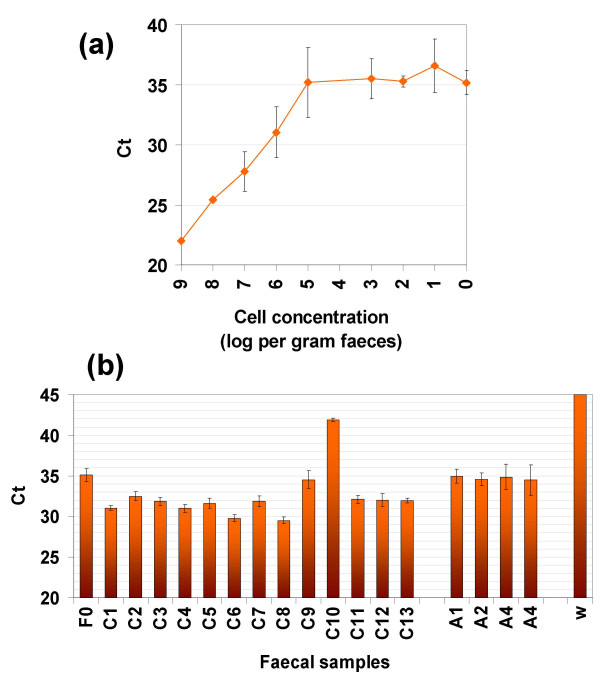
**Real-time PCR on faecal DNA samples**. (a) Ct values of spiked samples plotted against spiked cell concentration in faecal DNA sample. (b) Ct values obtained for children (C1-C13) and adult (A1-A4) faecal samples. F0: faecal sample free of DNA. w: water instead of DNA. Values represented are means and standard deviations for 3 repetitions.

The designed primers and probe allowed specific amplification of a 100-bp fragment located within the *pedA*-gene. As determined with spiked samples, the reaction was linear for concentrations ranging from 10^9 ^to 10^5 ^of *P. acidilactici *UVA1 cells per g of spiked faeces, corresponding to Ct values comprised between 22 and 35 (Figure 4(a)). A Ct value of 35 was thus fixed as the upper limit for detection. Samples with a higher value were considered not to contain any *pedA*-gene. With this assay, the pediocin gene was detected in DNA isolated from 11 out of 13 children faecal samples tested, but in none of the 4 adult samples (Figure 4(b)).

## Discussion

Data obtained from phenotypic characterization indicated that strain UVA1 is a *Pediococcus acidilactici*. The carbohydrate fermentation profile matched that described for *P. acidilactici *in the APILAB+ database and in the literature [8]. Furthermore, among *Pediococcus *species, only *P. acidilactici *is able to grow at 50°C [8]. It is currently accepted that the phenotypic characterization of a strain is not enough to discriminate species or even strains and a polyphasic approach for species identification is recommended [28]. The genotypic characterization using 16S rDNA sequence analysis confirmed the data from the phenotypic characterization, with strain UVA1 showing more than 99 % similarity to the 16S sequences of other *P. acidilactici *strains. Mora *et al*. 2000 [29] used restriction fragment length polymorphism (RFLP) of housekeeping genes and 16S rDNA sequence analysis to distinguish between pediocin- and non-pediocin-producing *P. acidilactici *strains and showed that the pediocin-producing strains represented a homogenous subpopulation. This is supported by our observation that strains UVA1 and UL5, two pediocin-producing *Pediococcus acidilactici*, are very closely related in regard to 16S sequence similarity.

*P. acidilactici *UVA1 inhibited *L. ivanovii *HPB28 by production of a proteinaceous compound, as demonstrated by the total loss of activity after proteolytic treatments. This compound had a molecular weight of approximately 4.5 kDa, similar to pediocin PA-1. This was confirmed by the detection in strain UVA1 of a plasmid of approximately 9.5 kb, on which we could localize the *pedA*-gene. We also found that the pediocin operon, which consists of the pediocin structural gene (*pedA*), the specific immunity gene (*pedB*), and genes required for processing, maturation and secretion of the bacteriocin (*pedC *and *D*), showed 99 to 100 % similarity to the published sequences for the pediocin PA-1/AcH operon [24,27]. In all pediocin-producing strains isolated so far, the genetic determinants are plasmid-encoded [5]. The comparison of genetic determinants of many pediocin-like producer strains [30] showed that all pediocin genes were carried by plasmids, but the surrounding sequences on the plasmids can differ from one strain to the other. Sequencing data for the regions upstream the regulatory region and downstream the transcription terminator of the pediocin operon in UVA1 suggested that the plasmid harboured by *P. acicilactici *UVA1 is probably identical to pSRQ11, a 9.4 kb plasmid from *P. acidilactici *PA-1 [24].

Transcription analysis by reverse-transcription PCR using primers specific for the mRNA transcript of a particular protein is a straightforward method to establish the link between the presence of the genetic determinant and the observed protein activity. With this direct method, we could show that the *pedA*-transcript was synthesized in exponentially growing cells of UVA1 but not in the cured, non-active derivative, bac-, and that the inhibitory activity of UVA1 was due to pediocin production. It is also interesting to notice that in *P. pentosaceus *DSM 20336^T^, the *pedA*-gene and the mRNA transcript were detected, but no inhibitory activity was observed against *L. ivanovii *HPB28 and the PCR with primers pedopF and pedopR did not yield any product (data not shown). Diep *et al*. [31] reported similar observations in *P. pentosaceus *ATCC 25745, where a truncated *pen *locus lead to low expression of antimicrobial activity.

Pediococci are commonly associated with various plants and their products or meat [8,32].) There are only few reports on *pediococci *detected in human faeces or gastrointestinal tract [12,33,34], and only Millette *et al*. [12], reported the presence of an antibacterial compound of proteinaceous nature, although not identified yet. Strain UVA1 is, to our knowledge, the first pediocin-producing *Pediococcus *to be isolated from human faecal material, which characteristic could be particularly interesting for the food industry for biopreservation as well as for possible probiotic effect. In this work, we showed, with a new real-time PCR assay, that strains containing the *pedA*-gene are relatively widespread in baby faecal material, but were not found in the 4 adult samples tested. Therefore, human faecal material could be a good source for isolating new pediocin-producing strains.

## Conclusion

Strain UVA1, isolated from new born faeces, was clearly identified as a *Pediococcus acidilactici *using a polyphasic approach. Data for the molecular size, sensitivity to protease and sequence of the plasmid-borne genetic determinant indicated that the antibacterial compound produced by UVA1 is pediocin PA-1/AcH. To our knowledge, *Pediococcus acidilactici *UVA1 is the first pediocin-producing *Pediococcus *strain isolated from human faeces. Real-time PCR was an efficient method for detection of specific genes in faecal samples harbouring a complex microbiota. We showed a large distribution of *pedA*-containing strains in baby faecal samples.

## Authors' contributions

SM participated in the study design and carried out the identification of the bacteriocin and the genetic study. UVA contributed to the study design, isolated and identified the strain UVA1 and participated in the writing of the manuscript. ES contributed to the characterisation of the strain. RM performed the real-time PCR screening of faecal samples. TC participated in the identification of the genetic determinant. LM and CL provided guidance during all parts of the work. All authors read and approved the final manuscript.
